# A rapidly progressing Pancoast syndrome due to pulmonary mucormycosis: a case report

**DOI:** 10.1186/1752-1947-5-388

**Published:** 2011-08-17

**Authors:** Meghana Bansal, Sara R Martin, Stacy A Rudnicki, Kim M Hiatt, Eduardo Mireles-Cabodevila

**Affiliations:** 1 Department of Internal Medicine, University of Arkansas for Medical Sciences, Little Rock, AR, USA; 2 Division of Pulmonary and Critical Care Medicine, University of Arkansas for Medical Sciences, Little Rock, AR, USA; 3 Division of Neurology, University of Arkansas for Medical Sciences, Little Rock, AR, USA; 4 Department of Pathology, University of Arkansas for Medical Sciences, Little Rock, AR, USA

## Abstract

**Introduction:**

Pancoast syndrome is characterized by Horner syndrome, shoulder pain radiating down the arm, compression of the brachial blood vessels, and, in long-standing cases, atrophy of the arm and hand muscles. It is most commonly associated with lung carcinoma but rarely is seen with certain infections.

**Case presentation:**

We present the case of a 51-year-old Caucasian man who had acute myeloid leukemia and who developed a rapidly fulminating pneumonia along with signs and symptoms of acute brachial plexopathy and left Horner syndrome. Also, a purpuric plaque developed over his left chest wall and progressed to skin necrosis. The skin biopsy and bronchoalveolar lavage showed a *Rhizopus *species, leading to a diagnosis of mucormycosis. This is a rare case of pneumonia due to mucormycosis associated with acute Pancoast syndrome.

**Conclusions:**

According to our review of the literature, only a few infectious agents have been reported to be associated with Pancoast syndrome. We found only three case reports of mucormycosis associated with acute Pancoast syndrome. Clinicians should consider mucormycosis in their differential diagnosis in a patient with pulmonary lesions and chest wall invasion with or without neurological symptoms, especially in the setting of neutropenia or other immunosuppressed conditions. It is important to recognize this condition early in order to target therapy and interventions.

## Introduction

Pancoast syndrome is most commonly associated with a primary lung carcinoma and rarely with metastatic malignancies and certain infections, including mucormycosis. It is characterized by Horner syndrome, shoulder pain radiating down the arm, compression of the brachial blood vessels, and, in long-standing cases, atrophy of the arm and hand muscles. Pulmonary mucormycosis usually occurs in the setting of hematological malignancies. The typical presentation is a patient with neutropenic fever, pulmonary infiltrates, and a clinical course that worsens despite antibiotics. We present a case of pulmonary mucormycosis associated with postchemotherapy neutropenia in a patient with acute myeloid leukemia. His course was fulminating, leading to chest wall invasion, brachial plexopathy, and Horner syndrome.

## Case presentation

A 51-year-old Caucasian man was hospitalized for the management of a relapse of acute myeloid leukemia. He had hyperleukocytosis and complained of mild shortness of breath and generalized weakness. He denied cough, fevers or chills, hemoptysis, or orthopnea. He had smoked 39 pack years. His significant medical history began six months prior to admission, when his condition was diagnosed as acute myelomonocytic leukemia. He had been treated with cytarabine and daunorubicin followed by high-dose cytarabine. Shortly after admission, he required urgent leukopheresis because of worsening hyperleukocytosis and acute respiratory failure. He recovered and had further chemotherapy with clofarabine and cytarabine.

**Figure 1:**
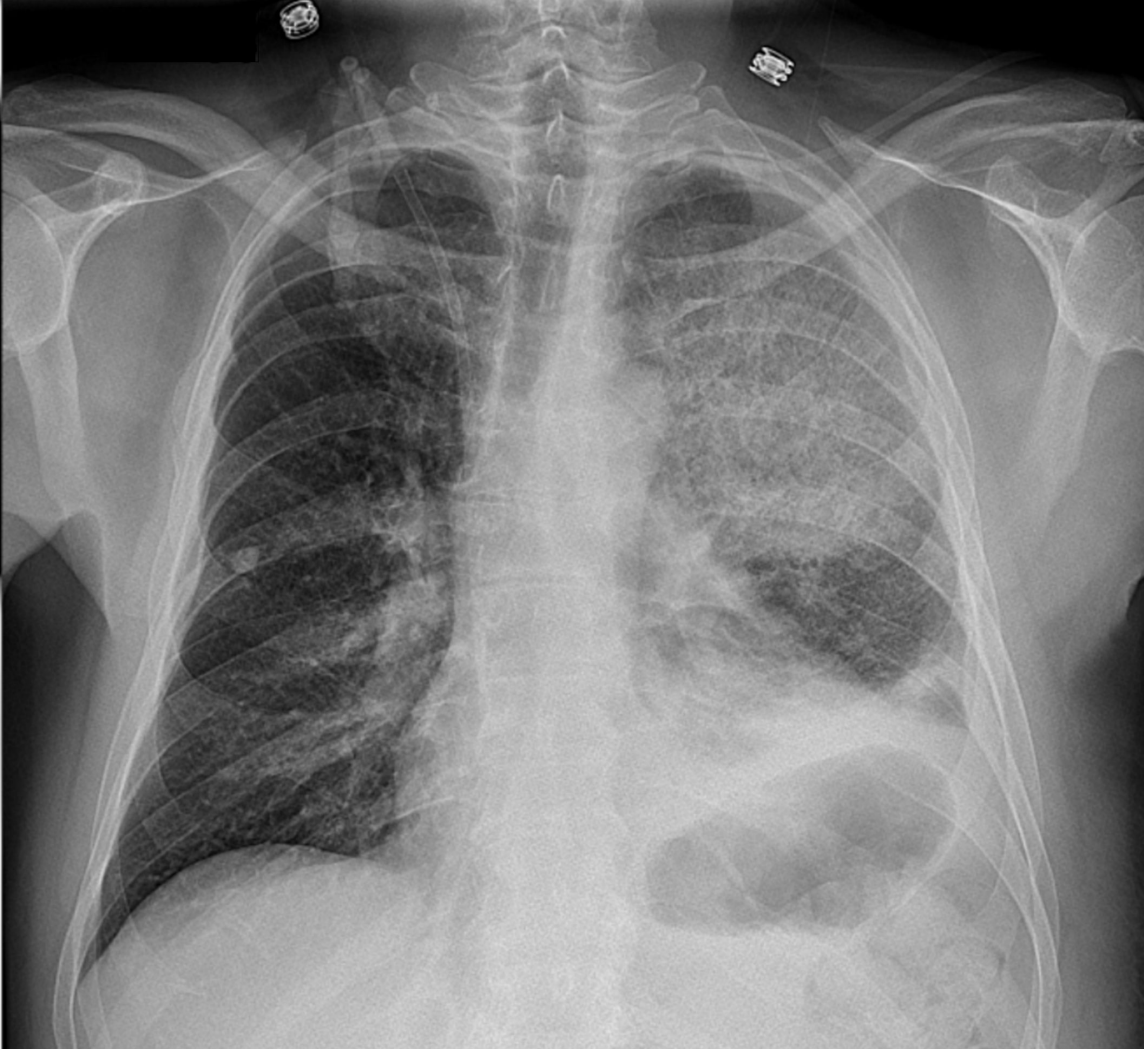
**A chest radiograph shows elevation of the left hemidiaphragm, alveolar and interstitial opacities of the left upper lobe and lower lobe with air bronchograms, and blunting of the costophrenic angle**.

Eighteen days after admission, he was neutropenic and febrile. He developed left axillary burning pain, which evolved in a matter of hours to numbness of his arm. Soon afterward, weakness started in his hand and later moved proximally up his arm. At that time, he had a temperature of 38°C, a respiratory rate of 20 breaths per minute, a heart rate of 133 beats per minute, blood pressure of 117/77 mm Hg, and an oxygen saturation of 94% on 50% venturi mask. He was dyspneic and a chest examination revealed signs of lung consolidation in the left upper and left lower lobes. A chest radiograph (Figure 1) revealed elevation of the left hemidiaphragm, alveolar and interstitial opacities of the left upper lobe and lower lobe with air bronchograms, and blunting of the costophrenic angle. He was started on broad-spectrum antibiotics and voriconazole prophylaxis.

**Figure 2:**
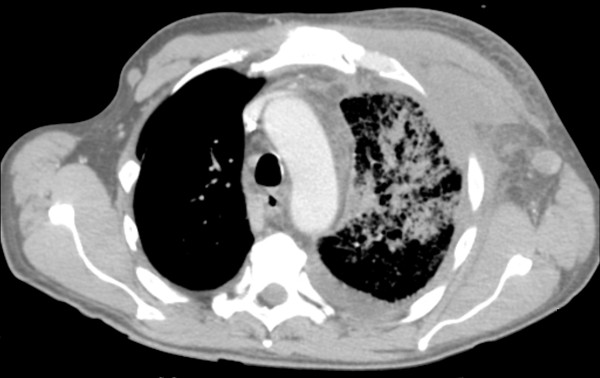
**Chest computed tomography (CT) image shows extensive edematous changes of the left chest wall and axilla, left pleural effusion, and pulmonary parenchymal consolidation**.

A physical examination revealed edema of the skin over the anterior left chest wall but no erythema, scaling, or discrete skin lesions. In a matter of hours, the numbness spread from a T1 distribution to a C8 distribution. The following day, his left arm was plegic with the exception of trace movement of the deltoid muscle. He was anesthetic to all sensory modalities in the left arm as well as areflexic. He also developed a left Horner syndrome. Further swelling developed in his left arm and anterosuperior chest with fullness in the supraclavicular fossa. Doppler ultrasound showed occlusion of his left distal subclavian, axillary, and brachial arteries and deep vein thrombosis of his left internal jugular, subclavian, and axillary veins. Heparin was started for his thrombosis, but soon after he had mild hemoptysis. A chest computed tomography scan (Figure 2) showed extensive edematous changes of the left chest wall and axilla along with left pleural effusion and pulmonary parenchymal consolidation. A bronchoscopy that revealed a blood clot in the left upper lobe bronchus without endobronchial lesions was performed.

**Figure 3:**
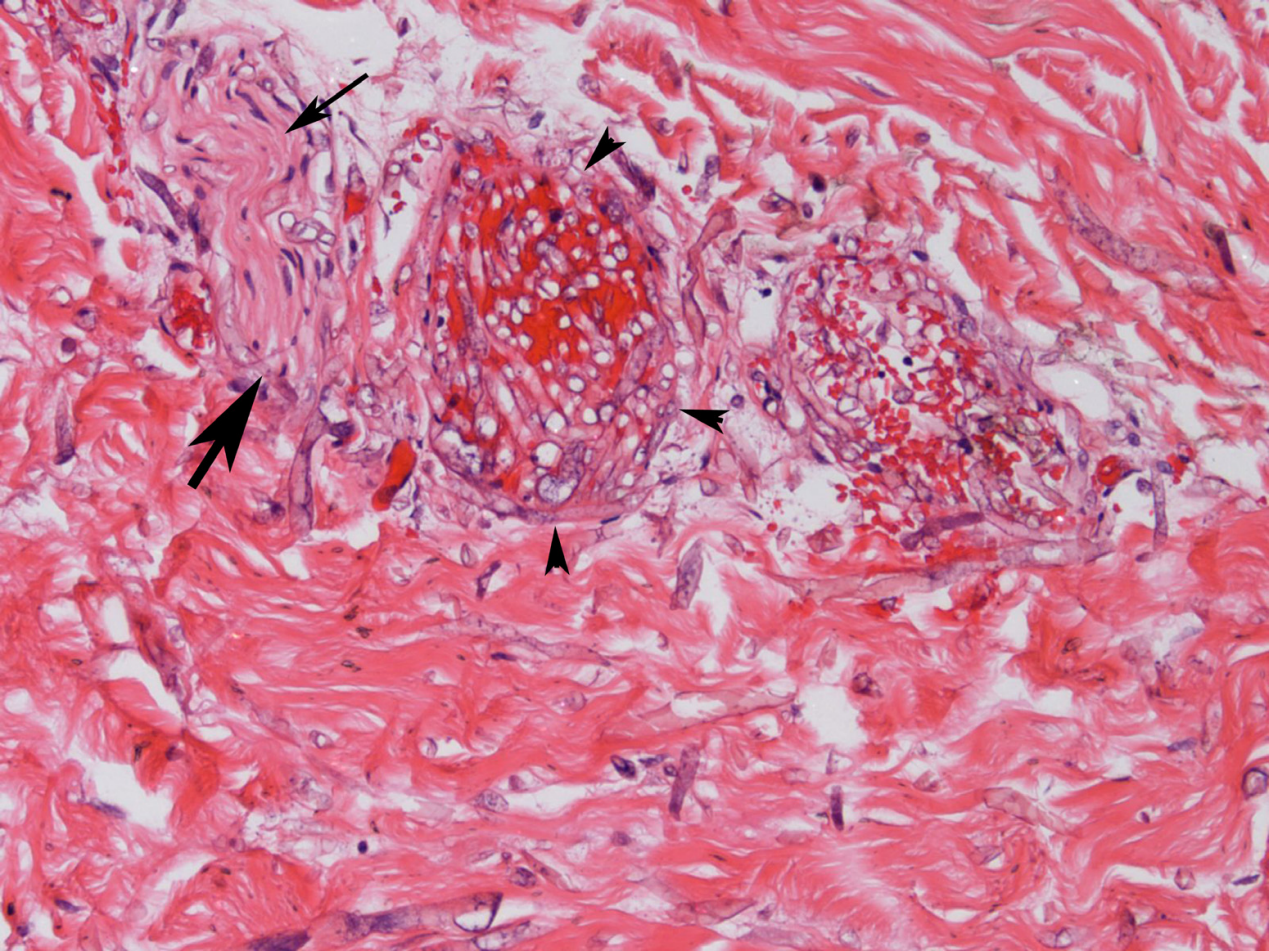
**Skin biopsy shows invasive fungi (large arrow) in the nerve (small arrow)**. The vessel (arrowheads) is filled with hyphae that are also seen traversing the vessel wall. Numerous hyphae are seen in the surrounding dermis as well (hematoxylin and eosin stain).

**Figure 4:**
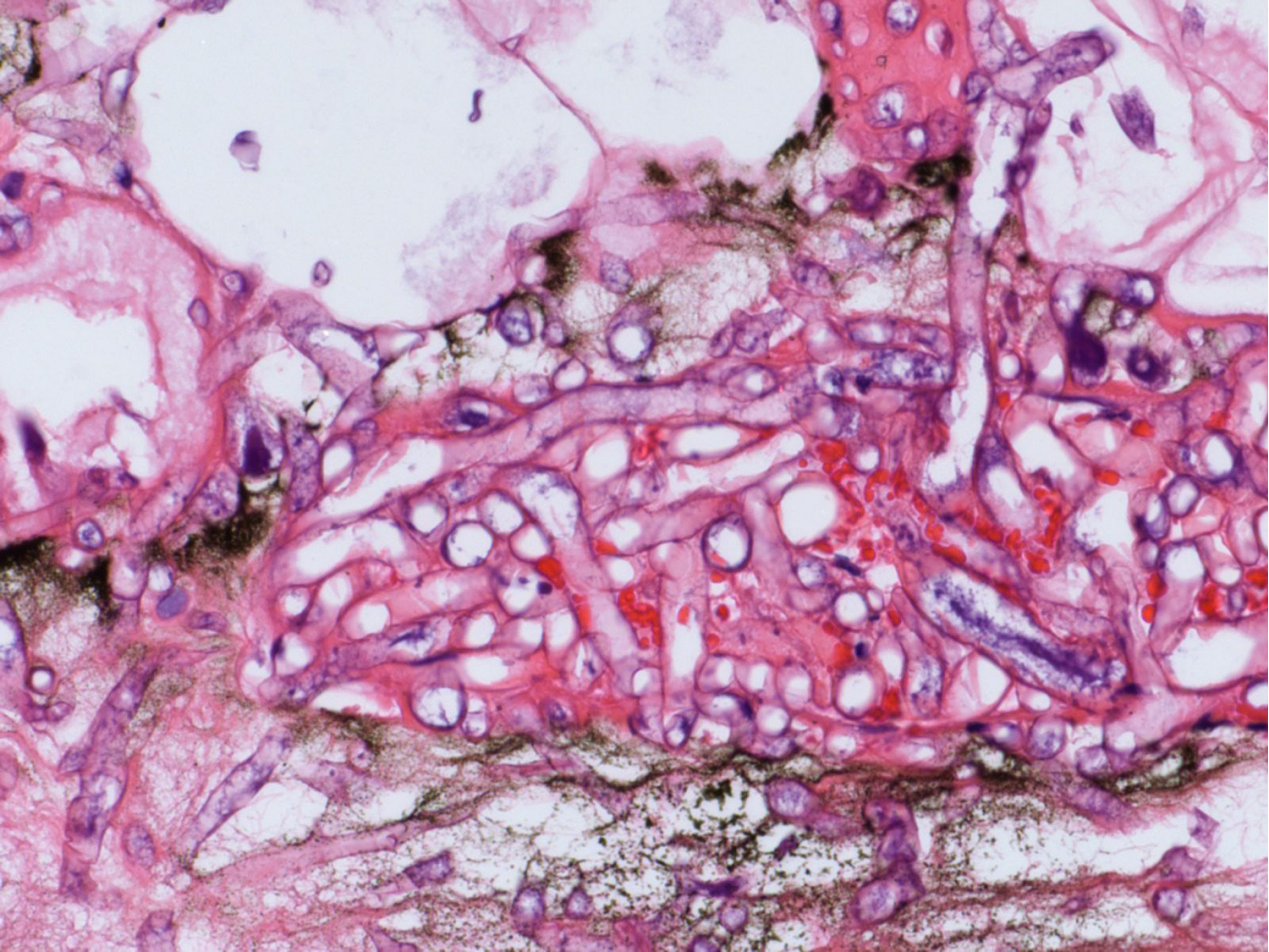
**Skin biopsy shows numerous fungi with broad hyphae, with right-angle branching (hematoxylin and eosin stain)**.

Six days after the onset of numbness, a palpable purpuric plaque developed over his left chest and progressed to a 2 cm black eschar. A punch biopsy of the plaque was performed. With failure to respond with broad spectrum antibiotics (which included vancomycin, imipenem, voriconazole and acyclovir), liposomal amphotericin B 5 mg/kg per day was added empirically to his regimen. A histological evaluation of skin biopsy showed abundant angiocentric and angioinvasive ribbon-like hyphae with irregular nonparallel contours, occasional right-angle branching, and rare septae (Figures 3 and 4). Cultures from both skin and bronchoalveolar lavage confirmed a diagnosis of mucormycosis by a *Rhizopus *species. Twenty-four hours later, he developed respiratory distress, shock, and multiorgan failure resulting in death. An autopsy was declined by his next of kin.

## Discussion

Mucormycosis is an uncommon opportunistic infection, but its incidence is increasing as the number of patients with diabetes, organ transplants, or immunosuppressed conditions continues to rise [1]. Hematological malignancies are more frequently associated with mucormycosis than solid tumors are [1]. In patients with hematological malignancies, mucormycosis affects the lungs most frequently (58% to 81%) [2].

Pulmonary mucormycosis occurs with inhalation of spores into the bronchioles and alveoli, usually resulting in a rapidly progressive pneumonia or endobronchial disease. Symptoms are typically nonspecific and may include fever, dyspnea, cough, and chest pain [2,3]. The infection can spread to contiguous organs or disseminate hematogenously. Of particular interest to our case is the overwhelming speed with which the process extended from the lung parenchyma to the surrounding structures (tissue, nerves, vessels, and skin). The progression of symptoms suggests that the lower brachial plexus trunk became ischemic initially and that the rest of the plexus followed as the lesion extended from the lung to the surrounding tissues. Mucor is known to locally invade nerves, most often in rhinocerebral disease. Common clinical findings include rhinitis, periorbital and facial swelling, mucosal necrosis, ophthalmoplegia, multiple cranial nerve palsies, facial pain, and headache [4]. Whereas stroke from rhinocerebral mucormycosis causing occlusion of the cavernous portion of the internal carotid is well documented [4], involvement of the brachial vascular and nervous plexus is a very unusual presentation. Three reports document mucormycosis presenting as a Pancoast syndrome [5-7]. Notably, two had diabetes (both survived) as their only risk factor and one had acute lymphoblastic leukemia (died).

Pancoast syndrome is characterized by Horner syndrome (ptosis, miosis, hemianhidrosis, and enophthalmos), shoulder pain radiating toward the axilla or ulnar aspect of the arm, compression of the brachial blood vessels, and atrophy of the arm and hand muscles. This syndrome is most commonly caused by a bronchogenic carcinoma in the superior sulcus of the lung with destructive lesions of the thoracic inlet and invasion of the brachial plexus and cervicothoracic sympathetic chain. Other causes include metastatic neoplasms and infections. In a recent review of 31 patients with infectious causes of Pancoast syndrome, five were immunocompromised (acute myelogenous leukemia, acute lymphoblastic leukemia, and postchemotherapy), and all had opportunistic organisms (*Aspergillus *spp., mucor, nocardia, and *Pseudoallescheria boydii*) [7]. In contrast, only one of the 26 immunocompetent patients had an opportunistic organism.

In immunocompromised patients, it can be challenging to distinguish pulmonary disease caused by mucormycosis from that caused by aspergillosis. The concomitant presence of sinusitis or a history of voriconazole prophylaxis may suggest mucormycosis [8,9]. Of note, our patient received voriconazole prophylaxis. The radiographic presentation of pulmonary mucormycosis can be diverse, and there are no pathognomonic radiographic features. In a review of imaging findings in 32 cases of mucormycosis, two thirds of the patients had consolidation as a main finding [10]. Other radiographic manifestations include cavitation, mass-like lesions, widened mediastinum, an "air crescent sign", "halo sign", pleural effusion, and fistula to the chest wall [10,11]. Chamilos and colleagues [8], in a retrospective chart review, found that multiple nodules (at least 10) and the presence of pleural effusion were more often found in mucormycosis than in aspergillosis.

Definitive diagnosis of mucormycosis often evades initial noninvasive techniques such as sputum and blood cultures. The organism may be recovered by bronchoscopy with bronchoalveolar lavage or transbronchial lung biopsy. However, because of the focal distribution of the disease, transthoracic computed tomography-guided needle biopsy or open lung biopsy may be required to make the diagnosis in pulmonary disease [1,12,13]. Histology is characterized by infarction and necrosis of tissue which results from an invasion of the vasculature by hyphae. The fungus can also be associated with neural, vascular, and cutaneous invasion, as in our patient. The fungus is usually recognized by broad, irregular, nonseptate, right-angled, branching hyphae and is demonstrated by hematoxylin and eosin and specialized fungal stains. In contrast, *Aspergillus *hyphae are narrow with septate branches at 45° angles. As with other fungal diseases, mucorales rarely grow by culture.

Treatment of mucormycosis involves a combination of surgical debridement of involved tissues and antifungal therapy with varying degrees of success, as noted in the literature [1,14,15]. Amphotericin B is the first-line antifungal treatment; delays in instituting therapy may be associated with increased mortality. Elimination of predisposing factors for infection, such as hyperglycemia, metabolic acidosis, and neutropenia, is critical. Pulmonary mucormycosis carries a very high mortality rate (60% to 90%) [1,14].

## Conclusions

It is important to consider the diagnosis of mucormycosis in a patient with pulmonary lesions and chest wall invasion with or without neurological symptoms, especially in the setting of neutropenia or other immunosuppressed conditions. Our patient had a rapid progression to multiorgan failure and refractory shock despite being treated with amphotericin B and died within two days of his diagnosis.

## Consent

Written informed consent was obtained from the next of kin for publication of this case report and any accompanying images. A copy of the written consent is available for review by the Editor-in-Chief of this journal.

## Competing interests

The authors declare that they have no competing interests.

## Authors' contributions

MB reviewed the literature and wrote a first draft of the manuscript and submitted the manuscript. SRM reviewed the literature and edited the manuscript. SAR reviewed the literature and wrote the neurological features and discussion. KMH wrote the pathological discussion and edited the manuscript. EM-C reviewed the literature and corrected and finalized the manuscript. All authors read and approved the final manuscript.
